# Microbiome variation in corals with distinct depth distribution ranges across a shallow–mesophotic gradient (15–85 m)

**DOI:** 10.1007/s00338-016-1517-x

**Published:** 2017-01-10

**Authors:** Bettina Glasl, Pim Bongaerts, Nathalie H. Elisabeth, Ove Hoegh-Guldberg, Gerhard J. Herndl, Pedro R. Frade

**Affiliations:** 1 Department of Limnology and Bio-Oceanography, University of Vienna, Althanstrasse 14, 1090 Vienna, Austria; 2 College of Science and Engineering, Australian Institute of Marine Science, AIMS@JCU, James Cook University, Townsville, Australia; 3 CARMABI, Piscaderabaai z/n, PO Box 2090, Willemstad, Curaçao; 4 Global Change Institute, The University of Queensland, St. Lucia, QLD 4072 Australia; 5 ARC Centre of Excellence for Coral Reef Studies, The University of Queensland, St. Lucia, QLD 4072 Australia; 6 Department of Marine Microbiology and Biogeochemistry, NIOZ Royal Netherlands Institute for Sea Research, Utrecht University, PO Box 59, 1790 AB Den Burg, The Netherlands; 7 Centre of Marine Sciences (CCMAR), University of Algarve, Campus of Gambelas, 8005-139 Faro, Portugal

**Keywords:** Mesophotic coral ecosystems, Prokaryotic community, 16S rRNA tag sequencing, Indicator species

## Abstract

Mesophotic coral ecosystems (MCEs) are generally poorly studied, and our knowledge of lower MCEs (below 60 m depth) is largely limited to visual surveys. Here, we provide a first detailed assessment of the prokaryotic community associated with scleractinian corals over a depth gradient to the lower mesophotic realm (15–85 m). Specimens of three Caribbean coral species exhibiting differences in their depth distribution ranges (*Agaricia grahamae*, *Madracis pharensis* and *Stephanocoenia intersepta*) were collected with a manned submersible on the island of Curaçao, and their prokaryotic communities assessed using 16S rRNA gene sequencing analysis. Corals with narrower depth distribution ranges (depth-specialists) were associated with a stable prokaryotic community, whereas corals with a broader niche range (depth-generalists) revealed a higher variability in their prokaryotic community. The observed depth effects match previously described patterns in *Symbiodinium* depth zonation. This highlights the contribution of structured microbial communities over depth to the coral’s ability to colonize a broader depth range.

**Electronic supplementary material:**

The online version of this article (doi:10.1007/s00338-016-1517-x) contains supplementary material, which is available to authorized users.

## Introduction

Mesophotic coral ecosystems (MCEs) represent an extension of shallow-water coral reefs and provide an extensive habitat for light-dependent corals in subtropical/tropical regions starting at 30–40 m and reaching down to depths of about 150 m (Lesser et al. 2009, Hinderstein et al. 2010). MCEs can be further divided into the “upper mesophotic” (30–60 m) and “lower mesophotic” (>60 m), with the first representing a transition zone between shallow-water and lower mesophotic communities (sharing species of opposing depth zones) and the latter representing a more specialized coral community (Bongaerts et al. 2010, 2015b; Kahng et al. 2010). Given the increased difficulty in accessing lower mesophotic depths, most studies have thus far been limited to visual surveys of the benthos, and consequently, little is known about the coral-associated microbial communities.

A recent molecular study demonstrated that lower mesophotic depths in the southern Caribbean harbor a genetically distinct coral and associated *Symbiodinium* community, likely reflecting specialization of both symbiotic partners to the mesophotic environment (Bongaerts et al. 2015b). The coral holobiont, however, harbors not only symbiotic phototrophic zooxanthellae but also numerous other microorganisms such as other protists, fungi, bacteria, archaea and viruses (Rohwer et al. 2002; Carlos et al. 2013). Prokaryotes, including bacteria and archaea, are of particular interest due to their diverse metabolic and functional capabilities within the coral holobiont and their potential to complement the metabolic needs of the coral host (Ainsworth et al. 2015; Thompson et al. 2015). Studies on shallow-water communities have shown that prokaryotic communities associated with corals are species specific (Rohwer et al. 2002) and have a significant influence on host resilience (Glasl et al. 2016). Another study has found coral microbiomes in *Seriatopora hystrix* to correlate to reef habitat (depth) and geographical location, but not to intrinsic factors such as host genetic lineage and *Symbiodinium* genotype (Pantos et al. 2015). Although the interest in coral–prokaryote interactions has increased over the last decade (reviewed by Thompson et al. 2015), only sparse information on the prokaryotic community associated with corals from the upper mesophotic is available (reviewed by Olson and Kellogg 2010) and data from lower MCEs are virtually non-existent (but see Ainsworth et al. 2015), despite the potential metabolic contribution of these communities (compared to *Symbiodinium*) to the energetic balance of corals given the extremely low light conditions at lower mesophotic depths (reviewed by Thompson et al. 2015).

Here, we provide a first assessment of the variation in the structure and composition of coral-tissue-dwelling prokaryotic communities from shallow reef habitats down to lower mesophotic depths in three common Caribbean coral species with broad, but distinct, depth distributions on the island of Curaçao. In this study, we aimed at (1) determining whether the lower mesophotic corals host a distinct prokaryotic community or indicator assemblages and (2) addressing the respective roles of depth and host in prokaryotic community structure.

## Materials and methods

Coral specimens of *Agaricia grahamae* (Wells, 1973), *Madracis pharensis* (Heller, 1868) and *Stephanocoenia intersepta* (Lamarck, 1836) were collected in March–April 2013 at two locations on the leeward side of the island of Curaçao—Buoy 0/1 and Seaquarium—as part of the Catlin Seaview Survey. Fragments of coral specimens were collected over their natural occurring depth distribution [*A. grahamae* from 55 m (±5 m) and 85 m (±5 m) depth, *M. pharensis* from 15 m (±5 m), 55 m (±5 m) and 85 m (±5 m) depth and *S. intersepta* from 15 m (±5 m) and 55 m (±5 m) depth] using the manned submersible “Curasub” operated by the Substation Curaçao or SCUBA (see Bongaerts et al. 2015b for further details). *Agaricia grahamae* and *S. intersepta* could not be collected at the shallower and deeper sampling depths, respectively, due to their limited depth distribution ranges. Small fragments (<4 cm^2^) were subsampled from each specimen before being flash-frozen in liquid nitrogen and stored at −80 °C until further processing. The remaining subsamples were cleaned with commercial bleach solution, rinsed in fresh water and dried to confirm species identity (Bongaerts et al. 2015b). Only specimens confirmed as the targeted three species were included in this study (see Table 1 for a summary of sample distribution).

**Table 1:** Overview of the number of collected samples, the number of retrieved sequences, alpha diversity (Shannon index), richness, evenness and richness estimations (Chao index) of the prokaryotic community associated with each of three studied coral species (*Agaricia grahamae, Madracis pharensis* and *Stephanocoenia intersepta*) over their natural depth distribution on the island of Curaçao

Species	Depth	Number of samples	Number of sequences	Shannon index	Richness	Evenness	OTUs in total	Chao index
*Agaricia grahamae*	15	–	–	–	–	–	–	–
	50–60	9	2824 ± 2037	2.81 ± 0.77	49 ± 15	0.72 ± 0.15	127	193
	80–90	4	3286 ± 3502	2.77 ± 0.35	45 ± 5	0.73 ± 0.08	80	108
*Madracis pharensis*	15	4	3570 ± 4106	3.11 ± 0.22	60 ± 4	0.76 ± 0.04	113	154
	50–60	12	6730 ± 6682	2.99 ± 0.56	55 ± 16	0.75 ± 0.10	173	216
	80–90	8	6686 ± 5891	3.09 ± 0.70	58 ± 14	0.76 ± 0.14	141	176
*Stephanocoenia intersepta*	15	6	1842 ± 1823	2.35 ± 0.60	41 ± 9	0.63 ± 0.14	105	142
	50–60	8	4223 ± 5345	2.96 ± 0.71	56 ± 20	0.74 ± 0.12	147	183
	80–90	–	–	–	–	–	–	–

Data were generated with a rarified operational taxonomic unit (OTU) table based on family level and under the exclusion of chloroplast sequences

## Sequencing and data analysis

Thawed samples were rinsed with Milli-Q water to remove loosely attached bacteria from the coral’s surface. After scraping the tissue off the skeleton using sterile scalpels, genomic DNA of 51 coral tissue samples was extracted using the FastDNA SPIN Kit for Soil (MP Biomedicals) and a 728-bp fragment of the 16S rRNA gene (amplified by primers U341F and U1053R) sequenced by IMGM using 454 GL FLX + technology (Roche). Barcoded sequence reads were de-noised in Acacia (version 1.52.b0; Bragg et al. 2012) and analyzed using QIIME (version 1.9.0; Caporaso et al. 2010b) following the protocol described in detail by Glasl et al. (2016). In brief, sequences were picked and clustered into operational taxonomic units (OTUs) based on >98% sequence similarity using USEARCH (version v5.2.236; Edgar 2010), checked for chimera, and singletons excluded. Representative sequences were picked and aligned with PyNAST (version 1.2.2; Caporaso et al. 2010a) using the Greengenes database (version 13.5). The taxonomy was assigned with the Ribosomal Database Project Classifier (version 2.2; Wang et al. 2007). Furthermore, chloroplast reads were removed and 409 sequences were randomly picked for each sample to compensate for different sequencing efforts. The rarefied OTUs were grouped at the family level, and their relative abundances per sample were used for statistical analyses in R (R Development Core Team 2008). Data were not normalized prior to multivariate analyses as no linear methods were applied. Demultiplexed 16S rRNA gene raw reads are available in the NCBI SRA database under accession number SRP092218.

The influence of depth and host species on the alpha diversity (Shannon–Weaver index), richness and evenness of the coral-tissue-associated prokaryotic community was assessed with analysis of variance (ANOVA). Canonical correspondence analysis (CCA) was used to determine whether location, depth and host species drive the prokaryotic community assemblage. The significance of those factors was verified using an ANOVA-like permutation test based on 9999 permutations.

Non-metric multidimensional scaling ordination (nMDS) based on quantitative and binary Bray–Curtis dissimilarities of relative abundance and presence/absence data (Anderson et al. 2006), respectively, was used to visualize the variation of the prokaryotic community among different host species for a single depth (at 55 m) and within each host species over their depth distribution. Differences in the community structure were tested by applying a permutational multivariate ANOVA (PERMANOVA) using Bray–Curtis dissimilarity matrices (Anderson et al. 2006). The homogeneity of multivariate dispersions was tested using a resemblance-based permutation test (PERMDISP).

Indicator values (IndVal) analysis (De Cáceres and Legendre 2009) was applied to identify prokaryotic families significantly associated (*p* < 0.05, when both specificity and fidelity have probabilities >0.5) with coral host species and depth zones.

## Results and discussion

Prokaryotic communities associated with the tissue of the corals were structured according to coral species and depth (permutation test CCA; *p* < 0.001 and *p* < 0.01, respectively; Electronic Supplementary Material, ESM, Table S1). As sampling location did not influence prokaryotic community composition, samples from both locations (ESM Table S2) were merged for further analysis. There were no significant differences in alpha diversity, richness or evenness of the prokaryotic communities (based on prokaryotic families) among the three studied coral species or in response to depth (within each species; ESM Tables S3, S4, S5). Consequently, the community composition, rather than alpha diversity, is responsible for the observed variation in prokaryotic communities across sampling depths and among host species.

**Fig. 1:**
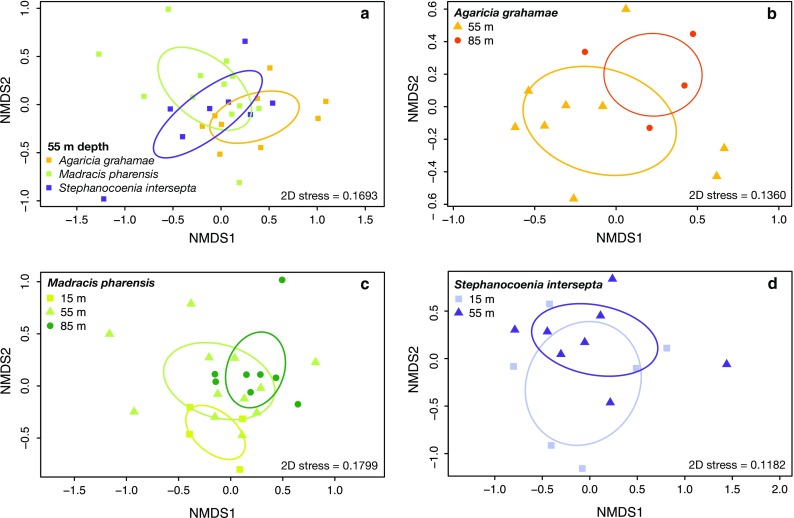
Non-metric multidimensional scaling ordination visualizing the prokaryotic community structure based on relative abundance of prokaryotic families (**a**) among the three different host species for a single depth (at 55 m) (**b, c, d**) within *Agaricia grahamae, Madracis pharensis* and *Stephanocoenia intersepta,* respectively, over their natural depth range (at 15, 55 and 85 m)

Tissue-associated prokaryotic community composition differed significantly (PERMANOVA, *p* < 0.05; Tables S6, S7) among *A. grahamae*, *M. pharensis* and *S. intersepta* at a single depth (55 m), which seems driven primarily by the large differences between *A. grahamae* and *M. pharensis* (Fig. 1a). Similar results (not shown) were obtained when the analysis was carried out with presence/absence data. These results are consistent with the widespread host specificity of prokaryotic community composition over space and time (Rohwer et al. 2002).

There were no significant differences in the prokaryotic community assemblage of *A. grahamae*, a deep-water specialist (Fig. 1b), between upper and lower depth populations (55 vs. 85 m; ESM Tables S8, S9). In contrast, *S. intersepta*, a depth-generalist (Fig. 1c), showed significant difference between depth zones, but only with presence/absence data (PERMANOVA, *p* < 0.01; ESM Tables S10, S11). This suggests that the rare or low-abundance prokaryotic families are driving the differences between depths within this species. Finally, *M. pharensis,* an “extreme” depth-generalist (Fig. 1d), showed significant depth variation (PERMANOVA, *p* < 0.01; ESM Tables S12, S13). The shallow (15 m) and the deep (85 m) populations of *M. pharensis* significantly varied in their prokaryotic community composition (PERMANOVA, *p* < ,0.01; ESM Table S14). *Madracis pharensis* samples from 55 m, however, seem to overlap with communities originating from the depth extremes and may therefore represent a transition from shallow water to the lower mesophotic reef. Similar results were obtained with presence/absence data (except for *S. intersepta*). Overall these results suggest that “depth-specialist” hosts, characterized by their restricted depth distribution (Bak 1977; Bongaerts et al. 2010), maintain a specific holobiont community. “Depth-generalist” hosts with a wide depth distribution (Bak 1977; Bongaerts et al. 2010), however, might host the most favorable prokaryotic composition for the surrounding environment (as also shown by Pantos et al. 2015). We hypothesize that this association of corals with a range of different prokaryotes over depth greatly contributes to host distribution and survival across the different depth habitats.

**Fig. 2:**
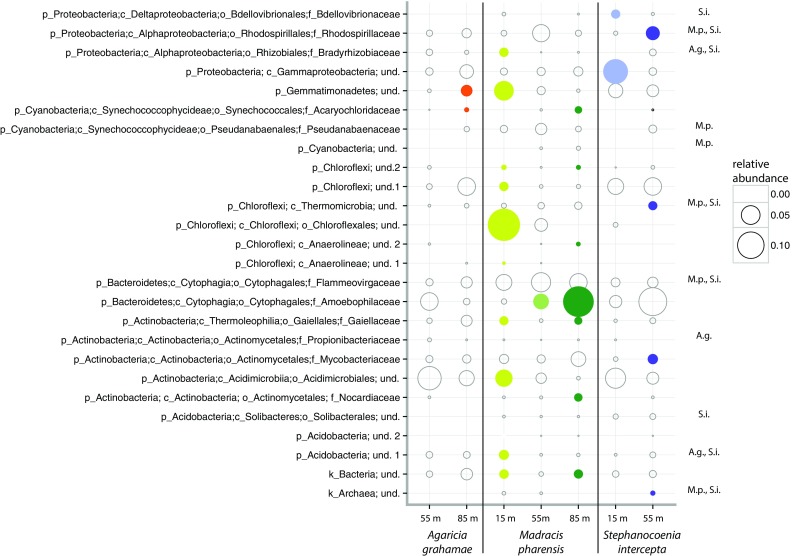
Relative abundance of indicator prokaryotic taxa associated with *Agaricia grahamae* (*A.g.*)*, Madracis pharensis* (*M.p.*) and *Stephanocoenia intersepta* (*S.i.*) at 55 m and among sampling depths (15, 55 and 85 m) for each individual coral species. Indicator taxa were identified with indicator values analysis to be significantly (*p* < 0.05) associated with a certain sampling host or depth group (indicated by *colored circles*)

Overall no single prokaryotic family was identified as universal depth indicator across all studied coral species (Fig. 2). Thus, the prokaryotic community seems to be generally shaped by its host rather than by predominant external environmental parameters (here represented by depth). However, depth-indicator prokaryotic taxa were identified within each individual coral species. *Agaricia grahamae* and *S. intersepta* hosted in total two and six prokaryotic taxa, respectively, that were identified as depth indicators (Fig. 2). In contrast to the species with more restricted depth distributions, *M. pharensis* harbored 14 prokaryotic taxa that were significantly associated with at least one particular depth zone (Fig. 2). For example, bacteria of the order Chloroflexales were significantly (IndVal; *p* < 0.05) associated with *M. pharensis* at 15 m depth, showed a steep decrease in their relative abundance toward the upper mesophotic and were totally absent in the lower mesophotic. The bacterial family Amoebophilaceae showed the opposite trend, with an increase in relative abundance with depth within *M. pharensis*, and were identified as a significant indicator (IndVal; *p* < 0.05) at 55 and 85 m depth. This is the first time that this bacterial family of known obligate intracellular amoeba symbionts (Schmitz-Esser et al. 2010) is recognized as an ecologically relevant member of the coral intratissue microbiome, an observation warranting further investigation. Overall, there is also a tendency for a higher relative abundance of cyanobacteria with increasing depth. This increase could relate to a modulation of *Symbiodinium* vs *Cyanobacteria* populations in holobionts reliant on photosynthetic input for nutrition (Lesser et al. 2007). Although further prokaryotic indicator taxa can be seen in Fig. 2, their exact function and metabolic potential remain elusive and require further investigation.

The observed spatial dynamics in prokaryotic community composition over depth for these three studied coral species closely resemble those reported earlier for their dominant *Symbiodinium* communities (as determined within the detection limits of ITS2-DGGE by Bongaerts et al. 2015a). *Agaricia grahamae*, for example, neither exhibits zonation in the genetic lineages of its associated *Symbiodinium* (type C3/C11 is ubiquitous, Bongaerts et al. 2015a) nor in the prokaryotic community over depth. In contrast, *M. pharensis* shows both a significant shift in the *Symbiodinium* community (type B7 in the shallow layers is completely replaced by type B15 in the mesophotic habitats; Bongaerts et al. 2015a) and in its prokaryotic community composition. Finally, *S. intersepta* exhibits a shift in the *Symbiodinium* community over its depth range (mixed communities changing from C16 to C3 and C1 as dominant types with increasing depth; Bongaerts et al. 2015a), as well as a shift in its prokaryotic community (based on presence/absence data). Although we cannot decisively differentiate between the effect of depth and the effects of the holobiont itself, there is evidence that the holobiont (*Symbiodinium* and/or coral host) modulates the prokaryotic community associated with the coral tissue (Ainsworth et al. 2015). This conclusion is in contrast with that of Pantos et al. (2015) for *Seriatopora hystrix*; they suggested that the variation in microbial communities associated with coral hosts is primarily driven by external environmental conditions. However, our study focused on the detection of prokaryotes associated with relatively stable intratissue microenvironment, whereas Pantos et al. (2015) likely included a large portion of coral surface mucus, whose associated prokaryotic communities are more exposed to the ambient reef environment and, therefore, more likely to vary spatially.

This study provides the first detailed assessment of the prokaryotic community associated with multiple scleractinian corals toward the lower mesophotic reef. The depth distribution range of coral species seemed to affect the overall variability of the prokaryotic community associated with coral tissue. Coral species with narrower depth distribution ranges retained a stable prokaryotic community, whereas corals with a broader depth distribution revealed higher taxonomic flexibility in their associated prokaryotic community. The observed depth effects are consistent with earlier published *Symbiodinium* variation (Bongaerts et al. 2015a). This highlights the contribution of structured microbial communities over depth to the coral’s ability to colonize a broader depth range.

## Electronic supplementary material

Below is the link to the electronic supplementary material.

Supplementary material 1 (DOCX 46 kb)
