# Supplementary material for: Microbiome variation in corals with distinct depth distribution ranges across a shallow–mesophotic gradient (15–85 m)
Source: Coral Reefs. 2017 Jan 10;36(2):447–52. doi: 10.1007/s00338-016-1517-x (PMC5434129; doi:10.1007/s00338-016-1517-x)
Supplement: Supplementary file 1 — Supplementary material 1 (DOCX 46 kb) [file 338_2016_1517_MOESM1_ESM.docx]

**Table S1** Statistical output of an ANOVA-like permutation test based on 9,999 permutations depicting significance of factors such as sampling location, sampling depth and coral species on the prokaryotic community associated with corals. *** p < 0.001, ** p < 0.01

|  | **df** | **χ^2^** | **F** | **Number of permutations** | **Pr(>F)** |
| --- | --- | --- | --- | --- | --- |
| Location | 1 | 0.1078 | 1.0734 | 9999 | 0.3281 |
| Depth | 1 | 0.2166 | 2.1573 | 9999 | 0.0013 ** |
| Species | 2 | 0.3478 | 1.7319 | 9999 | 0.0002 *** |
| Residual | 47 | 4.7187 |  |  |  |

**Table S2** Number of samples collected at different reef locations. Conspecific samples were merged for further analyses, as location did not have a significant effect on the prokaryotic community assemblage

| **location** | ***Agaricia grahamae*** | ***Madracis pharensis*** | ***Stephanocoenia intersepta*** |
| --- | --- | --- | --- |
| Buoy 0/1 | 6 | 12 | 6 |
| Seaquarium | 7 | 12 | 8 |
| Combined locations | 13 | 24 | 14 |

**Table S3** Statistical output of ANOVA used to test differences in alpha diversity (Shannon index) between the different sampling groups (depth and species)

|  | **df** | **SS** | **MS** | **F value** | **Pr(>F)** |
| --- | --- | --- | --- | --- | --- |
| Sample group | 6 | 2.516 | 0.4194 | 1.056 | 0.403 |
| Residuals | 44 | 17.473 | 0.3971 |  |  |

**Table S4** Statistical output of ANOVA used to test differences in richness between the different sampling groups (depth and species)

|  | **df** | **SS** | **MS** | **F value** | **Pr(>F)** |
| --- | --- | --- | --- | --- | --- |
| Sample group | 6 | 1760 | 293.4 | 1.43 | 0.225 |
| Residuals | 44 | 9031 | 205.2 |  |  |

**Table S5** Statistical output of ANOVA used to test differences in evenness between the different sampling groups (depth and species)

|  | **df** | **SS** | **MS** | **F value** | **Pr(>F)** |
| --- | --- | --- | --- | --- | --- |
| Sample group | 6 | 0.0747 | 0.01245 | 0.851 | 0.538 |
| Residuals | 44 | 0.6438 | 0.01463 |  |  |

**Table S6** Statistical output of a permutation-based analysis (PERMDISP) testing homogeneity of multivariate dispersions between *Agaricia grahamae*, *Madracis pharensis* and *Stephanocoenia intersepta* at a single depth (55 m)

|  | **df** | **SS** | **MS** | **F value** | **Pr(>F)** |
| --- | --- | --- | --- | --- | --- |
| Species 55 m | 2 | 0.021770 | 0.010885 | 1.563 | 0.2527 |
| Residuals | 11 | 0.076604 | 0.006964 |  |  |

**Table S7** Statistical output of permutational multivariate analysis of variance (PERMANOVA) testing differences in community composition between *Agaricia grahamae*, *Madracis pharensis* and *Stephanocoenia intersepta* for a single depth (55 m). * p < 0.05

|  | **df** | **SS** | **MS** | **F model** | **R2** | **Pr(>F)** |
| --- | --- | --- | --- | --- | --- | --- |
| Species 55 m | 2 | 0.6284 | 0.31421 | 1.3428 | 0.19623 | 0.04496 * |
| Residuals | 11 | 2.5740 | 0.23400 |  | 0.80377 |  |
| Total | 13 | 3.2024 |  |  | 1.00000 |  |

**Table S8** Statistical output of a permutation-based analysis (PERMDISP) testing homogeneity of multivariate dispersions between the different sampling depths (55 vs. 85 m) of *Agaricia grahamae*

|  | **df** | **SS** | **MS** | **F value** | **Pr(>F)** |
| --- | --- | --- | --- | --- | --- |
| Depth | 1 | 0.034406 | 0.034406 | 3.7072 | 0.0782 |
| Residuals | 12 | 0.111373 | 0.009281 |  |  |

**Table S9** Statistical output of permutational multivariate analysis of variance (PERMANOVA) testing differences in community composition between the different sampling depths (55 vs. 85 m) of *Agaricia grahamae*

|  | **df** | **SS** | **MS** | **F model** | **R2** | **Pr(>F)** |
| --- | --- | --- | --- | --- | --- | --- |
| Depth | 1 | 0.3149 | 0.31486 | 3.7072 | 0.09506 | 0.1918 |
| Residuals | 12 | 2.9974 | 0.24978 |  | 0.90494 |  |
| Total | 13 | 3.3123 |  |  | 1.00000 |  |

**Table S10** Statistical output of a permutation-based analysis (PERMDISP) testing homogeneity of multivariate dispersions between the different sampling depths (15 vs. 55 m) of *Stephanocoenia intersepta*

|  | **df** | **SS** | **MS** | **F value** | **Pr(>F)** |
| --- | --- | --- | --- | --- | --- |
| Depth | 1 | 0.003187 | 0.0031866 | 0.4942 | 0.4955 |
| Residuals | 12 | 0.077371 | 0.0064476 |  |  |

**Table S11** Statistical output of permutational multivariate analysis of variance (PERMANOVA) testing differences in community composition between the different sampling depths (15 vs*.* 55 m) of *Stephanocoenia intersepta*

|  | **df** | **SS** | **MS** | **F model** | **R2** | **Pr(>F)** |
| --- | --- | --- | --- | --- | --- | --- |
| Depth | 1 | 0.4544 | 0.45443 | 1.7416 | 0.12674 | 0.07493 |
| Residuals | 12 | 3.1311 | 0.26093 |  | 0.87326 |  |
| Total | 13 | 3.5856 |  |  | 1.00000 |  |

**Table S12** Statistical output of a permutation-based analysis (PERMDISP) testing homogeneity of multivariate dispersions between the different sampling depths (15 vs. 55 vs. 85 m) of *Madracis pharensis*

|  | **df** | **SS** | **MS** | **F value** | **Pr(>F)** |
| --- | --- | --- | --- | --- | --- |
| Depth | 2 | 0.045142 | 0.0225709 | 3.115 | 0.06536 |
| Residuals | 21 | 0.152164 | 0.0072459 |  |  |

**Table S13** Statistical output of permutational multivariate analysis of variance (PERMANOVA) testing differences in community composition between the different sampling depths (15 vs. 55 vs. 85 m) of *Madracis pharensis.* ** p < 0.01

|  | **df** | **SS** | **MS** | **F model** | **R2** | **Pr(>F)** |
| --- | --- | --- | --- | --- | --- | --- |
| Depth | 2 | 0.7307 | 0.36535 | 1.7048 | 0.13968 | 0.005994 ** |
| Residuals | 21 | 4.5004 | 0.21430 |  | 0.86032 |  |
| Total | 23 | 5.2311 |  |  | 1.00000 |  |

**Table S14** Statistical output of pairwise permutational multivariate analysis of variance (PERMANOVA) testing differences in community composition between the different sampling depths (15 vs. 55, 55 vs. 85, and 15 vs. 85 m) of *Madracis pharensis* based on 10,000 permutations. ** p < 0.01

|  | M.p.15 | M.p.55 |
| --- | --- | --- |
| M.p.55 | 0.0805 | - |
| M.p.85 | 0.0066** | 0.0805 |
